# Saphenofemoral arteriovenous fistula as hemodialysis access

**DOI:** 10.1186/1471-2482-10-28

**Published:** 2010-10-18

**Authors:** João A Correa, Luiz Carlos de Abreu, Adilson C Pires, João R Breda, Yumiko R Yamazaki, Alexandre C Fioretti, Vitor E Valenti, Luiz Carlos M Vanderlei, Hugo Macedo Junior, Eduardo Colombari, Fausto Miranda

**Affiliations:** 1 Departamento de Cirugia da Faculdade de Medicina do ABC, Santo André, SP, Brasil; 2 Laboratório de Escrita Científica, Departamento de Morfologia e Fisiologia, Faculdade de Medicina do ABC, Santo André, SP, Brasil; 3 Departamento de Medicina, Disciplina de Cardiologia, Universidade Federal de São Paulo (UNIFESP), São Paulo, SP, Brasil; 4 Departamento de Fisioterapia, Universidade Estadual Paulista (UNESP), Presidente Prudente, SP, Brasil; 5 Disciplina de Cirurgia Cardiovascular, Universidade Federal de São Paulo (UNIFESP), São Paulo, SP, Brasil

## Abstract

**Background:**

An upper limb arteriovenous (AV) fistula is the access of choice for haemodialysis (HD). There have been few reports of saphenofemoral AV fistulas (SFAVF) over the last 10-20 years because of previous suggestions of poor patencies and needling difficulties. Here, we describe our clinical experience with SFAVF.

**Methods:**

SFAVFs were evaluated using the following variables: immediate results, early and late complications, intraoperative and postoperative complications (up to day 30), efficiency of the fistula after the onset of needling and complications associated to its use.

**Results:**

Fifty-six SFAVF fistulas were created in 48 patients. Eight patients had two fistulas: 8 patent (16%), 10 transplanted (20%), 12 deaths (24%), 1 low flow (2%) and 20 thrombosis (39%) (first two months of preparation). One patient had severe hypotension during surgery, which caused thrombosis of the fistula, which was successfully thrombectomised, four thrombosed fistulae were successfully thrombectomised and revised on the first postoperative day. After 59 months of follow-up, primary patency was 44%.

**Conclusion:**

SFAVF is an adequate alternative for patients without the possibility for other access in the upper limbs, allowing efficient dialysis with good long-term patency with a low complication rate.

## Background

The establishment of vascular access for hemodialysis (HD) remains a challenge for vascular surgery. Due to the improvement of chronic renal failure treatment and consequently, increased survival, maintenance of vascular access and long-term treatment of its complications, it has become an important cause of hospitalization, morbidity and patient costs [1,2].

The access of choice for HD is a primary arteriovenous (AV) fistula in the upper limb, between the radial artery and the cephalic vein [3-6]. This technique increases venous flow approximately 250-300 ml per minute, which is the minimum flow velocity needed to obtain the appropriate clearance of urea after 4 hours of HD. The preference for its use is due to the longer period of working of these AV communications, low rate of complications and easy handling [7-9]. Prolonged use of these accesses may lead to complications such as infection, puncture pseudoaneurysm, anastomotic pseudoaneurysm, venous hypertension, distal ischemia and their own obstruction [10]. In this situation, generally creation of new AV fistula is indicated.

Some disadvantages of using AV fistula by transposition of the femoral vein are the necessity for a more extensive and deeper dissection that uses larger vessels and make it more susceptible to infection and steal syndrome [11]. Such problems were also observed by us [12] and motivated us to seek a new alternative technique, using a saphenous vein in "bridge"; conformation, anastomosed to the superficial femoral artery near the adductor canal [12]. The initial benefits of this procedure encouraged us to expand the study with a larger patient number, analyzing the results of clinical experience and late complications. Therefore, in view of the above considerations, we aimed to evaluate the results of saphenofemoral AV fistula (SFAVF) as access to HD.

## Method

This is a longitudinal prospective study conducted at the Hospital of the Faculdade de Medicina do ABC, in São Bernardo do Campo (SP). The patients were from the Department of Nephrology, Faculdade de Medicina do ABC, São Bernardo do Campo (SP), and from the Nephrology and Hypertension Center, Santo André (SP). We included patients in whom all access possibilities were exhausted in the upper limbs and could not be treated by peritoneal dialysis, while those with an absent or inadequate long saphenous vein, sequelae of deep venous thrombosis or a femoropopliteal arterial occlusion were excluded.

This research was explained to all subjects and it was begun only after their consent according to the standards of the Ethic Committee in Research of our University (protocol number 021/2000). All patients gave informed consent. All procedures were in compliance with the Helsinki Declaration.

From August 1998 to May 2006 1183 fistulas for HD access were performed. 1086 were native (91.8%) and 97 (8.2%) were prosthetic polytetrafluoroethylene (PTFE). Among the 1086 native, 1030 (87.1%) were performed in upper limbs and 56 (4.7%) in lower limbs and used the saphenous vein. Among the fistulas which used prostheses, 79 (6.7%) were performed in upper limbs and 18 (1.5%) in lower limbs.

The 56 SFAVF were performed in 48 patients who underwent HD, in which all access possibilities were exhausted and limbs met the inclusion criteria. Data from each patient were recorded in a protocol for further statistical evaluation.

The sample consisted of 27 female (56.2%) and 21 male (43.7%) patients. In eight patients (6 women and 2 men) two SFAVF were performed, these were performed at the same time. Ages ranged from 10 to 82 years old (mean 44.4 years old). One 10-year old patient was of small stature and had no

One 10-year old patient was of small stature and had no vessels of an appropriate size. Among the 48 patients, 42 (87.5%) presented one or more associated diseases such as hypertension (HT), diabetes mellitus (DM), heart and arterial diseases.

We conducted analysis of cumulative patency by Kaplan-Meier method and other results were presented as a simple percentage.

The surgical technique consisted of making an AV fistula by using only one anastomosis between the saphenous vein and the distal superficial femoral artery after its implementation and superficial to the anterior thigh.

Patients were discharged on the first day after surgery after verification of clinical conditions and surgical outcomes. The follow-up was performed on an outpatient basis, every seven days in the HD unit. Thirty days later the fistula was released to punctures.

Fistulas were evaluated according to puncture, HD flow, spontaneous venous pressure absence and dialysis adequacy according to weekly Kt/v [13]. Puncture facility was defined as catheterization of the fistula at its first attempt, the minimal acceptable HD flow was considered as values above 250 ml/min; absence of spontaneous venous pressure was considered as pressure lower than 100/mm Hg at the end of devolution with optimal blood flow and urea clearance dialysis adequacy (in vitro) was multiplied by the duration of dialysis in minutes divided by the volume of urea distribution (weight × 0.6). We considered 1.2 as the ideal week value.

We also evaluated the presence or absence of complications such as thrombosis, post-puncture hematoma, distal limb ischemia, venous hypertension, cardiac decompensation, infection, puncture pseudoaneurysm, anastomotic pseudoaneurysm and aneurysmal dilatation. If a problem was found we used ultrasonography and Echo-color-Doppler 2P. In cases of stenosis we treated with an angioplasty and in cases of pseudoaneurysm we performed a surgical revision.

The immediate result was defined as thrill intensity and presence or absence of postoperative complications after surgery. Regarding fistula maturation we considered early outcome as the fistula maturation from the 1^st ^to the 30^th ^post-operative day and late result as the fistula maturation after punches onset (after the 30^th ^post-operative).

### Technical Standardization

**Figure 1:**
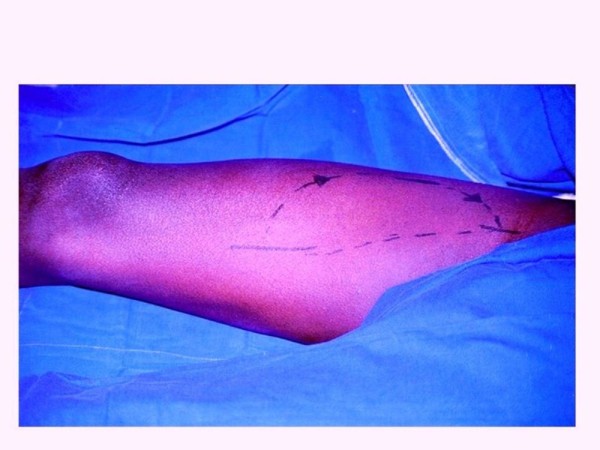
**Patient positioned for surgery with demarcation of the planning technique**.

**Figure 2:**
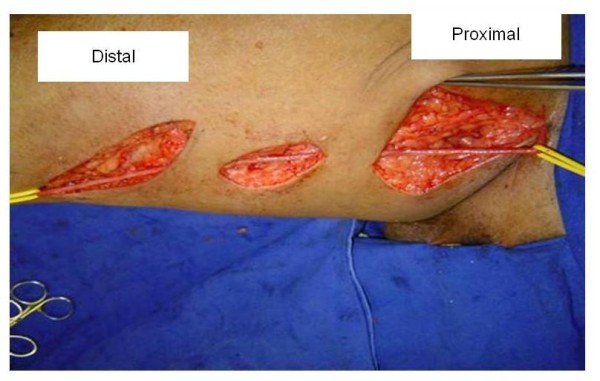
**Dissection of the saphenous vein through stab incisions, starting from the inguinal region to the distal third of thigh**.

**Figure 3:**
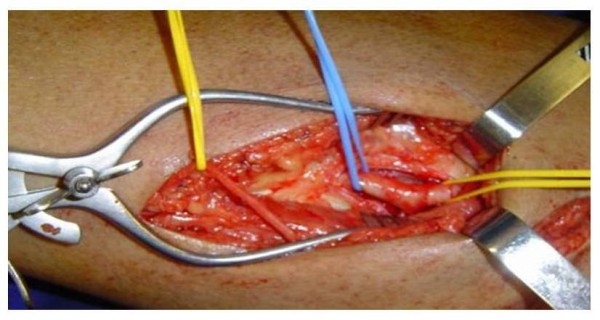
**Dissection of the superficial femoral artery above the adductor canal**.

**Figure 4:**
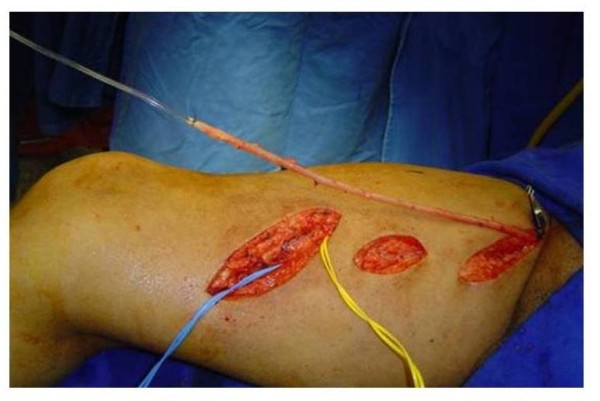
**Saphenous vein dissected, it was removed from its bed and was catheterized**.

**Figure 5:**
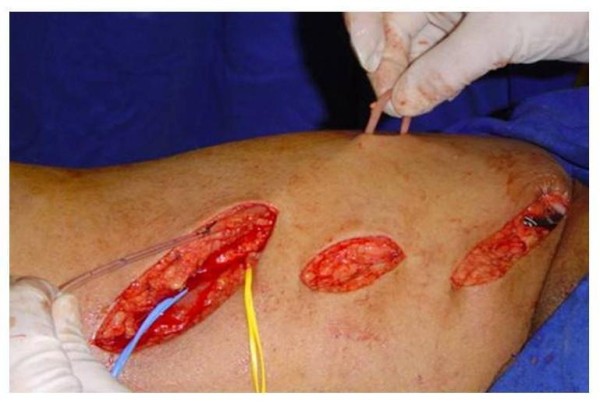
**Passage of the saphenous vein through the subcutaneous tunnel on the anterior thigh**.

**Figure 6:**
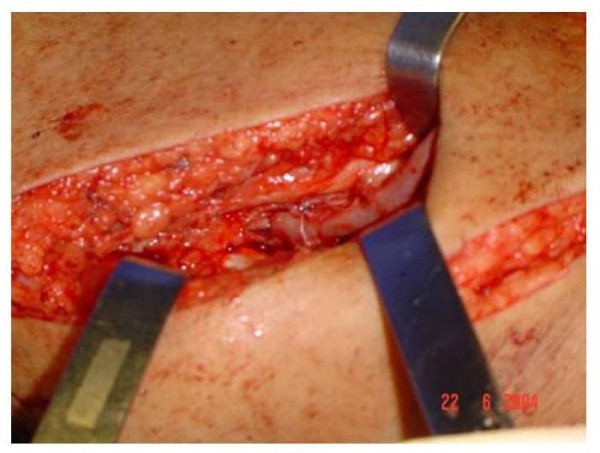
Anastomosis between the saphenous vein and superficial femoral artery

**Figure 7:**
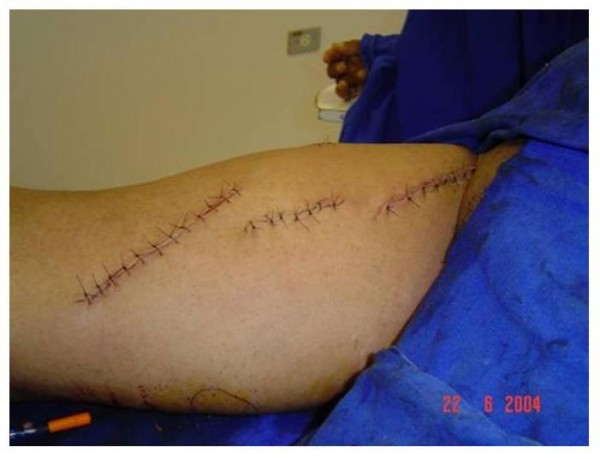
**Final aspect of the surgical scars**.

Patients were submitted to SFAVF creation according to the following standardization: (1) Election of the most suitable limb for manufacture of AV fistula through clinical examination based on arterial and venous conditions: presence of normal peripheral pulses and no signs of chronic venous hypertension or varicose vein disease. (2) Patient on horizontal dorsal supine position under spinal anesthesia. (3) Antisepsis with iodopovidine and delimitation of the surgical area with sterile cloths (Figure 1). (4) Longitudinal incision of approximately 6 cm in the inguinal region, dissection and ligation of tributary veins of the proximal saphenous vein. (5) Stab incisions for dissection of the saphenous vein to its distal third of the thigh (Figure 2). (6) Oblique incision of approximately 10 cm in the middle third of the distal inner thigh. (7) Dissection of the distal thigh followed by planes of dissection and isolation of the superficial femoral artery above the hiatus of the adductor magnus medially away from the sartorius muscle (Figure 3). (8) Distal ligation, removal of the bed with plastic tube catheterization number six and heparinization of the saphenous vein with 20 ml of heparin 1% (Figure 4). (9) Production of subcutaneous tunnel by blunt dissection in the anterolateral thigh in order to place the saphenous vein in front of its new bed. (10) Proximal and distal superficial femoral artery clamp and longitudinal arteriotomy of approximately 1 cm. (11) Proximal heparinization with 10 ml of heparin to 1%, end-to-side saphenous vein to the femoral artery, using running sutures of 6-0 polypropylene. (12) Arterial and venous unclamp according to maneuvers and pulmonary embolic protection. (13) Passage of the saphenous vein through the subcutaneous tunnel (Figure 5). (14) Verification of the final terms, as the conformation of the vein and the thrill of the proximal and distal AV fistula. Figure 6 represents the anastomosis between the saphenous vein and superficial femoral artery and Figure 7 presents the final aspect of the surgical scars.

## Results

Fifty-six fistulas were used in 48 patients, 27 females and 21 males. Six patients underwent construction of two saphenous vein fistulas (four females and two males). The oldest is in operation for 59 months and the youngest for three months. These fistulas were efficient, did not present difficulty to puncture, presented good HD flow (300 ml/min), presented no spontaneous venous pressure and dialysis adequacy according to kt/v (within the ideal value) [13].

Ten patients underwent renal transplantation with cadaver kidney (20.8% - seven females and three males). The age ranged from 24 to 51 years old (mean 34.7 years old). These transplants occurred from the 3^rd ^to the 37^th ^month after fistulas creation (average of 9.6 months) (Table 1).

**Table 1:** Distribution of SFAVF according to analysis after 93 months of follow-up (N = 51).

Development	Number of fistulas	%
Functioning	8	15.7
Transplanted	10	19.8
Deaths	12	23.5
Thrombosis	20	39.2
Low flow	1	1.9

% = Percentage.

N = Number.

In this follow-up of 59 months, among the 56 fistulas that were used in 48 patients we found four types of complications: thrombosis, pseudoaneurysm, stenosis/low flow and puncture hematoma. Thrombosis and low flow with functional loss occurred in 21 fistulas (41.2% - 11 were male and 10 female patients). The working period of the fistula ranged from two to 30 months (average of 9.5 months of use). The age ranged from 10 to 80 years old (average of 43.3 years old). Pseudoaneurysm puncture occurred in five fistulas (9.8% - all patients were female). The earliest occurred in four months and the latest occurred after 30 months of use. Stenosis with low flow fistulas occurred in two cases (5.9% - one male and one female) with a mean time of seven months. Puncture hematoma occurred in a fistula in a female patient at the 3^rd ^month of its maturation and its absorption occurred spontaneously without jeopardize the fistula patency. Other complications such as: ischemia, venous hypertension, infection, anastomotic pseudoaneurysm, aneurysmal dilatation and cardiac decompensation were not observed.

After treatment of complications, these fistulas could be used for a varied period until the following outcomes occured: maintenance of patency, transplantation, death or thrombosis.

In cases of patients with thrombosis or low flow (21 fistulas, eight subjects), it was decided to carry out SFAVF in the contralateral limb, and in two, both male, we decided to implant a PTFE in bridge, using the superficial femoral artery and common femoral vein of the contralateral limb. In another case, also male, we also decided by the PTFE to implant in "loop" using the common femoral artery and vein in the inguinal region in the same limb which the SFAVF was performed. In the same condition it was held biopsy of the saphenous vein thrombosis of the anterior fistula.

In other cases of thrombosis due to the urgent necessity for HD it was decided to install double-lumen catheter (perm-cath^®^) through the femoral vein in the inguinal region. In only two cases was it possible to perform peritoneal dialysis.

In 93 months of follow-up there were twelve deaths in patients who had their SFAVF able (eight females and four males), the youngest was 37 years old and the oldest 80 years old, with an average of 50 years. The maturation period of these fistulas ranged from two to 40 months, with an average of 16.6 months.

At this time eight fistulas continue to be used in four females and four males, the youngest aged 30 years old and the oldest aged 53 years old (average of 42.9 years old). The most recent has three months of operation and the oldest 59 months (average of 16 months). Since the beginning of the investigation until now, we have experienced eight patent fistulas, 10 transplant patients, 12 deaths, 20 thromboses and one low flow.

**Figure 8:**
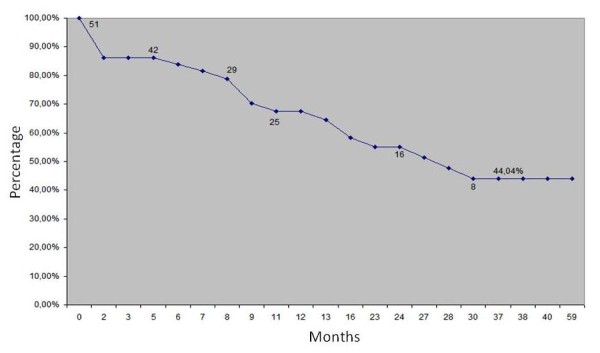
**Cumulative patency curve by the Kaplan-Meier method in 59 months (±5.49%; N = 51)**.

**Figure 9:**
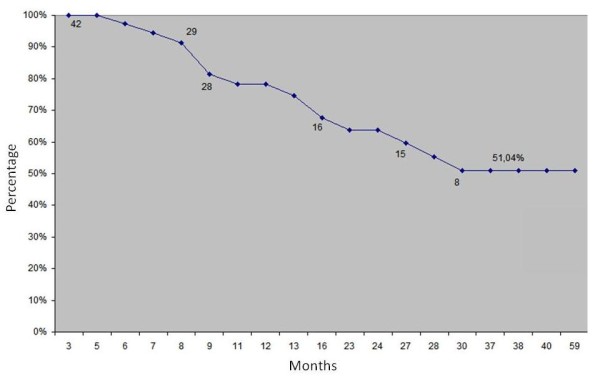
**Cumulative patency curve by the Kaplan-Meier method in 59 months excluding primary failures (±6.36%; N = 42)**.

We carried out the analysis of cumulative patency [14], with a score of 44.04% patency likely at the end of the period of 59 months, which is the duration of the oldest fistula operation. We observe in Figure 8 cumulative patency curve by the Kaplan-Meier method in 59 months and in Figure 9 we observe cumulative patency curve by the Kaplan-Meier method in 59 months excluding primary failures.

By eliminating the primary failure, i.e., fistulas thrombosis in the first two months of preparation, according to the recommendations of the National Kidney Foundation (NKF)-Dialysis Outcomes Quality Initiative (DOQI) Clinical Practice Guidelines for Vascular Access, 1997, it was withdrawn from analysis nine fistulas (seven thromboses and two due to death), we obtained then a patency of 51.04% in this period.

In all cases the primary (five pseudoaneurysms and three stenosis) and second (one thrombosis) patencies were maintained.

## Discussion

According to our findings, SFAVF was a viable option in patients without vascular access options in upper limbs and it presented good efficacy, low morbidity, complications which could be corrected and good patency rate in long term.

The choice of anastomosis site in the distal superficial femoral artery above the adductor canal was decided in order to avoid kinking of the saphenous vein during its accommodation in the subcutaneous tunnel [15] and also to keep it away from the inguinal region, where the risk of infection is higher [15]. Another advantage is that artery is easily accessible and makes it easier to perform anastomosis. In this technique, anastomosis is protected by the sartorius muscle, hence avoiding close punctures and decreasing the risk of anastomotic pseudoaneurysms.

The "bridge" technique with the superficial anterior saphenous vein offers facilities to punctures; it is a long vein segment, thus avoiding repeated punctures at the same location, which minimizes the risk of pseudoaneurysms, thrombosis and early stenosis [16]. Furthermore, based on our experience, it also enables the patient to a comfortable and safe position during HD.

The most serious and common complication in this study was thrombosis, however, when it was performed during the intraoperative period until the first postoperative day, whether by technical failure, severe hypotension or compression by hematoma due to coagulation disorders, it was corrected instantly through a new surgical approach restoring the fistula patency. This procedure was necessary in five fistulas in our study, one occurred in the intraoperative period and four on the first days after surgery, and these fistulas were used later.

At late follow-up, i.e. 30 days after fistula creation, among the 56 fistulas that were used there were 21 losses, 20 by thrombosis and reduced blood flow, with time course ranging from two to 30 months; the largest number of thrombosis occurred in the first two months (33.3%). The higher thrombosis concentration in this period reminds us that this fact is also observed in all other forms of native fistulas, especially in the wrist radio-cephalic AV fistula [17], which follows as the standard reference of vascular access for HD, and it presents a high rate of early or immediate failure, ranging from 10 to 30%, reaching some groups nearly 50% and it presents high risk for diabetics in elderly and women [18].

The losses caused by late thrombosis and decreased blood flow occurred in 14 fistulas, it probably occurred in many of these cases due to multiple punctures at the same location, leading to stenosis, low flow and subsequent thrombosis [17]. The suspicion of repeated punctures could be observed by clinical examination, where patients presented an area of skin nodules hypopigmentation and hard nodule in the vein segment at the corresponding region. In the pathological examination performed in a vein fragment of a patient it was found myointimal hyperplasia of the vein, which demonstrates thickening of venous wall.

We observed some cases of thrombosis due to prolonged arterial hypotension and one case due to trauma of the fistula site at home environment. By studying this type of complication, we alert to the importance of maintaining regular surveillance with periodic evaluation of these fistulas in order to detect early dysfunction so that it may be corrected in time, reducing the risk of thrombosis and increasing the usefulness period of the AV fistula [19].

Puncture pseudoaneurysm occurred in five fistulas (9.8%). It may be clinically diagnosed and confirmed by fistulography and ultrasound. This complication was probably due to inadequate compression of the puncture site after dialysis sessions associated with repeated punctures in one place, leading to vein wall weakness. Nonetheless, this type of complication was possible to be corrected in three cases. In two cases it was corrected through its resection and interposition of a new vein segment while in the third case it was corrected by resection and placement of a venous patch, maintaining the primary patency. Vigilance with regard to this complication should be intense. When skin is affected the risk of rupture is too large and should therefore be performed surgical correction as early as possible in those situations [20].

We observed one case of post-puncture hematoma due to compression and inadequate care after dialysis session. This fistula may be preserved through clinical treatment and by spontaneous absorption of the hematoma; however, the risk of an infection is always high in such cases. It may be necessary in some cases to perform surgical drainage.

Stenoses were detected by flow decrease during dialysis session. These situations could be confirmed by fistulography, through digital subtraction in three cases. This complication could be corrected in two cases by endovascular therapy through percutaneous transluminal dilatation, which maintained the fistula patency. Moreover, its use may be retaken one day after this procedure. Therefore, to correct stenosis with hemodynamic repercussion in this type of access as well as in other grafts access types it should be made as early as possible in order to keep an adequate dialysis to decrease thrombosis rate and consequently increase the survival.

When it is impossible to correct thrombosis, the creation of a new SFAVF in the contralateral limb may be indicated, since the saphenous vein and superficial femoral artery allow it. This option was used in eight patients in our investigation. In some cases of thrombosis, which the long saphenous vein of the contralateral limb was already used, it was necessary to use PTFE in bridge by using the superficial femoral artery and distal common femoral vein in the inguinal region. In case of saphenous vein absence, this was our first choice because there was facility to accommodate the prosthesis in subcutaneous tissue.

The option to implant a PTFE "in loop" using common femoral artery and vein in the inguinal region, as performed by Khadra et al [17] and Bhandari et al [16], was our last choice, we executed it in cases that it was already performed in the same SFAVF limb and the contralateral limb was unable to use the saphenous vein and to implant PTFE in bridge. In some cases we used this technical option to prevent fibrosis in the anterior region of the anastomosis between the superficial femoral vein and artery, thereby avoiding the creation of a new "bridge" fistula conformation. Notwithstanding, this technique presents high infection rate as reported by Bhandari et al [16], who presented infection range of 35% and Khadra et al [17], who showed infection rate of 16%.

Other complications such as distal ischemia, venous hypertension, cardiac decompensation, anastomotic pseudoaneurysm, aneurysmal dilatation, infection or other complications were not observed in our experience. Conversely, Taylor et al [22] performed 45 grafts ("in loop" and "in thigh"), in whom PTFE prosthesis were used in 39 cases and bovine carotid artery in six cases. They observed high rate of non-thrombotic complications with 18% of infection and 16% of distal limb ischemia.

A new analysis was done after exclusion of primary failure, i.e. the losses in the first two months of its creation, as recommended by various conduct guidelines for vascular access (NKF-DOQI, Spanish Society of Nephrology). This analysis showed that the rate increased to 51.04% in 60 months in 42 fistulas. On the other hand, analysis of results after 12 and 24 months revealed a patency rate of 78.2% and 63.8% respectively, a rate close to researches published by Brescia and Cimino [18], which primary patency at six months ranged between 65% and 81%.

The limitation of this technique is given in cases when the patient presents saphenous vein absence or when the saphenous vein is inadequate for this purpose and also in patients with arterial occlusive disease in the femoropopliteal territory. Another limitation of this technique is that the saphenous vein prevents the development of the fistula due to its developed muscle layer, similar to the cephalic vein in the internal forearm AV fistula. Although it prevents aneurysmal dilatation it increases the risk of myointimal hyperplasia after repeated punctures of the AV fistula [20]. Nevertheless, because it is autologous material, presents low cost, higher infection resistance and it is easy to handling, the advantages compensate its limitations even when compared to other access techniques in lower limbs which also uses autologous material such as transposition of the superficial femoral vein, first described by Huber et al [23], which reported two cases of use of this vein, one in the thigh and one in the arm and also reported by Gradman et al [24], in a retrospective study of 25 cases, which used this technique in lower limbs. This technique, which is an exception procedure, showed very good results in its long-term use according to our findings.

## Conclusion

Saphenofemoral arteriovenous fistula is a viable alternative in patients which present no more options for vascular access in upper limbs. It presents good efficacy, low morbidity, complications that may be corrected and good patency rate in long term.

## Competing interests

The authors declare that they have no competing interests.

## Authors' contributions

All authors participated in the acquisition of data and revision of the manuscript. JAC and FMJ conceived of the study, determined the design, performed the statistical analysis, interpreted the data and drafted the manuscript. All authors read and gave final approval for the version submitted for publication.

## Pre-publication history

The pre-publication history for this paper can be accessed here:

http://www.biomedcentral.com/1471-2482/10/28/prepub
